# Staff and Institutional Factors Associated with Substandard Care in the Management of Postpartum Hemorrhage

**DOI:** 10.1371/journal.pone.0151998

**Published:** 2016-03-24

**Authors:** A. Rousseau, P. Rozenberg, E. Perrodeau, C. Deneux-Tharaux, P. Ravaud

**Affiliations:** 1 Department of Obstetrics and Gynecology, Poissy-Saint Germain Hospital, Poissy, France; 2 INSERM U1153, METHODS (Méthodes en évaluation thérapeutique des maladies chroniques) Research Unit. Paris Descartes-Sorbonne Paris Cité University, Paris, France; 3 Research unit EA 7285, Versailles-St Quentin University, Saint Quentin en Yvelines, France; 4 Assistance Publique-Hôpitaux de Paris, Centre d’Epidémiologie Clinique, Hôpital Hôtel-Dieu, Paris, France; 5 INSERM U1153, EPOPé (Epidémiologie Obstétricale, Périnatale et Pédiatrique) Research Unit, Paris Descartes-Sorbonne Paris Cité University, Paris, France; Oslo University Hospital, Ullevål, NORWAY

## Abstract

**Objective:**

to identify staff and institutional factors associated with substandard care by midwives managing postpartum hemorrhage (PPH).

**Methods:**

A multicenter vignette-based study was e-mailed to a random sample of midwives at 145 French maternity units that belonged to 15 randomly selected perinatal networks. Midwives were asked to describe how they would manage two case-vignettes about PPH and to complete a short questionnaire about their individual (e.g., age, experience, and full- vs. part-time practice) and institutional (private or public status and level of care) characteristics. These previously validated case-vignettes described two different scenarios: vignette 1, a typical immediate, severe PPH, and vignette 2, a severe but gradual hemorrhage. Experts consensually defined 14 criteria to judge adherence to guidelines. The number of errors (possible range: 0 to 14) for the 14 criteria quantified PPH guideline adherence, separately for each vignette.

**Results:**

450 midwives from 87 maternity units provided complete responses. Perfect adherence (no error for any of the 14 criteria) was low: 25.1% for vignette 1 and 4.2% for vignette 2. After multivariate analysis, midwives’ age remained significantly associated with a greater risk of error in guideline adherence in both vignettes (IRR 1.19 [1.09; 1.29] for vignette 1, and IRR 1.11 [1.05; 1.18] for vignette 2), and the practice of mortality and morbidity reviews in the unit with a lower risk (IRR 0.80 [0.64; 0.99], IRR 0.78 [0.66; 0.93] respectively). Risk-taking scores (IRR 1.41 [1.19; 1.67]) and full-time practice (IRR 0.83 [0.71; 0.97]) were significantly associated with adherence only in vignette 1.

**Conclusions:**

Both staff and institutional factors may be associated with substandard care in midwives’ PPH management.

## Introduction

Severe postpartum hemorrhage (PPH) is a leading cause of maternal mortality and morbidity worldwide [1–3] and represents 1% to 2% of deliveries in high-income countries [3–5]. The incidence of PPH is increasing worldwide. [6–10] Hemorrhage accounts for 12% of pregnancy-related deaths in the United States [11,12] and 18% in France [13,14]. Moreover, reports from confidential enquiries revealed that 67% of those in the United States were preventable [15] and 85% in France [14,16], because they were due to delay in treatment or inadequate management.

Furthermore, important variations in clinical practice related to PPH occur between and within countries, despite relatively similar national guidelines [17–22].

Two kinds of factors may be considered to explain variations in practice and PPH severity: factors related to characteristics of women and deliveries, and factors related to their medical care [23,24]. Factors related to medical care involve characteristics of the staff and of the institution and probably play a significant role in the poor translation of guidelines into clinical practice. Furthermore, they may constitute modifiable features in health systems.

Farquhar et al [23] and Geller et al [15] identified contributory and avoidable factors of maternal deaths, including organizational and staff factors such as inadequate education and training, or lack of staff knowledge. Two other studies have reported less than optimal management of severe PPH and failure to apply guidelines fully in approximately 40% of cases [25,26], partly due to maternity unit status. These studies used retrospective medical records, however, and it is difficult to control for case mix in retrospective chart reviews. Furthermore, chart abstraction underestimates the quality of care [27].

Clinical vignettes have been widely used to compare quality of clinical care and to assess practice variation [24,28–30]. In a previous study, we demonstrated that dynamic vignettes with several steps are a valid and useful tool that can accurately reflect real practices in complex emergency situations, such as severe PPH [31]. Accordingly, the case-vignette method can be used to identify factors associated with variations in practice and to understand discrepancies between guidelines and practices in PPH management. In some countries, such as France and the United Kingdom, midwives diagnose and provide initial care for PPH, at the same time that they call for an obstetrician. Midwives are qualified to administer the first-line uterotonic agent (oxytocin) and to perform manual placental delivery, manual examination of the uterine cavity, uterine massage, monitoring, and initial resuscitation measures. They work closely with the obstetricians and anesthesiologists on duty to manage life-threatening situations, notably severe PPH. In a series of papers and comments, the *Lancet* recently demonstrated the contribution that midwifery can make to the quality of care of women and infants [32].

The objective of our study was to identify staff and institutional factors associated with substandard care in PPH management by midwives.

## Material and Methods

This multicenter cross-sectional study took place from January to April 2014. Our institutional review board (Comité de Protection des Personnes Ile de France Paris- XI) approved this study on September 13, 2012, as number 12066.

Midwives were given a link to dedicated website, where they were asked to complete this survey, describing how they would manage 2 case-vignettes about PPH and responding to a short questionnaire about individual staff and unit characteristics.

### Survey instrument: case-vignettes

In our previous validation study, we developed 66 dynamic case-vignettes describing incidents of severe PPH in several steps, based on documentation in patient files [31]. Briefly, vignettes were developed by abstracting the following data from patient files: patient medical history and information about the pregnancy, labor, delivery and PPH.

Two case-vignettes among the 66 were selected by six obstetrics professionals: three midwives and three obstetricians. They opted for two very different case-vignettes: vignette 1 described a typical immediate, severe PPH, and vignette 2 a less typical case of severe but gradual PPH with a constant trickle of blood (see Files in S1 and S2 Files).

These two vignettes described the postpartum course and included multiple-choice questions detailing proposed clinical care. We designed the vignettes to include three successive steps re-creating the course of the PPH. For the first step, we showed a partogram summarizing the medical history, labor, delivery and PPH at diagnosis. The second step of the vignette presented the postpartum course over the next 15 minutes (response to treatment) visually, and the third step the following 15 minutes: bleeding was illustrated by pictures of simulated soaked pads and containers [33], and maternal condition by pictures of a simulated monitor display (pulse, blood pressure, and SpO_2_). At each step, the midwives were asked how they would manage this emergency situation.

### Participants

**Fig 1:**
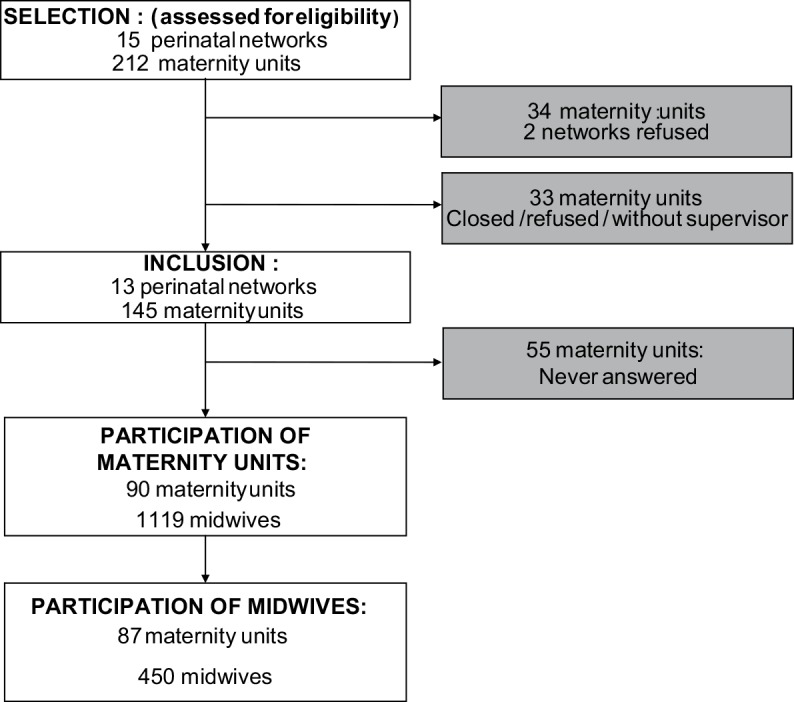
Flowchart. This figure depicts to the flowchart of the study.

We randomly selected 15 perinatal networks in France, to include about half the total number. All maternity units here, both public and private, belong to a perinatal network grouping together level-1 (no facilities for nonroutine neonatal care) and level-2 (with a neonatal care unit) units around one or more level-3 units (reference centers with an onsite neonatal intensive care unit). In all, the 215 maternity units of these 15 perinatal networks were eligible. Two entire perinatal networks (i.e., 37 maternity units) chose not to participate. Moreover, among the 13 networks that did participate, 33 maternity units either decided not to or closed before our study started. Therefore our sample included 145 maternity units, accounting for 27% of all French maternity units (Fig 1).

### Survey administration

We sent an email to the supervising midwife in each participating maternity unit, explaining the aim of the survey and asking her to transmit the link to the survey website by email to all midwives who worked during a arbitrarily selected period (from January 13 to 19, 2014, that is, Monday to Sunday) in the unit’s delivery room. Midwives were thus randomly selected within each maternity unit. If the midwives did not respond to the survey, their supervisor received two gentle email reminders, 2 weeks apart [34].

### Study variables

#### Outcome variables

For each step, we used the same multiple-choice questions with three different types of management, for the midwife to choose none, one, or more actions from the list of choices for each type of management (see Files in S1 Text):

- pharmacological management: antibiotic, oxytocin, misoprostol (prostaglandin E1 analogue), methylergometrine, sulprostone (prostaglandin E2 analogue), or tranexamic acid;
- non-pharmacological management: abdominal ultrasound, uterine massage, bimanual uterine compression, torsion of the cervix, bladder catheterization, manual examination of the uterine cavity, cervical examination with speculum, perineal repair, intrauterine tamponade, selective arterial embolization, or surgical treatment;
- communication, monitoring and investigation: alert other members of the team, venipuncture for blood sampling, resuscitation and monitoring

After answering the questions for each step, participants could not return to the previous step to change their answer.

Criteria for assessing responses were determined in a two-step procedure involving two separate expert committees. The first comprised 3 midwives and 3 obstetricians previously involved in developing French guidelines for PPH or conducting studies on this topic. They were asked to respond to the two vignettes according to guidelines published by the French College of Obstetricians and Gynecologists [21, 35], which are similar to those of both the American College of Obstetricians and Gynecologists [20] and the Royal College of Obstetricians and Gynaecologists [36]. A second committee of one obstetrician, one midwife, and one epidemiologist reviewed their answers and selected as criteria only those responses selected by all members of the first committee. Finally, 14 criteria were used to define adherence to guidelines for each vignette: 3 for pharmacological management, 8 for non-pharmacological management, and 3 criteria for other management (communication, monitoring, and investigation) (Table 1): some were answers that had to be chosen, while others were answers that were always wrong in that circumstance. The remaining responses were neither correct nor incorrect and did not count in the assessment.

**Table 1:** Criteria for evaluation of adherence to guidelines.

**Pharmacological management:**
First line uterotonic: oxytocin in step 1
Second line uterotonic: sulprostone (prostaglandin E2 analogue) in step 2
No misoprostol (prostaglandin E1 analogue) in each step
**Non-pharmacological management:**
Manual placental delivery, manual examination of the uterine cavity in step 1
No intrauterine tamponade in step 1
No torsion of the cervix in step 1
Uterine massage in steps 1 or 2
Cervical examination with speculum in steps 1 or 2
No surgical treatment in steps 1 or 2
No selective arterial embolization in steps 1 or 2
Surgical treatment, selective arterial embolization and/or intrauterine tamponade in step 3
**Communication, monitoring, and investigation:**
Alert other members of the team in steps 1 or 2
Venipuncture with blood count, hemostasis in steps 1 or 2
Resuscitation measure in steps 1 or 2

We assessed the number of errors among the 14 selected criteria to quantify adherence to guidelines (theoretical range of possible errors: 1–14). This adherence was assessed separately for each vignette (expected responses were, however, identical). Any errors, that is, failure to adhere the guidelines, was considered equivalent to providing substandard care.

#### Independent variables

Participants answered a short questionnaire about:

- personal factors: age, experience, gender, time in practice, risk-taking score (measured by a published 6-item risk attitude scale), and fear of malpractice (measured by a published 2-item fear-of-malpractice scale) [37,38]. Briefly, respondents were asked how strongly they agreed with 8 statements on a Likert scale ranging from strongly disagree to strongly agree.
- organizational factors in their maternity units: size (number of births per year), status (as private or public, university or non-university), level of neonatal care (level 1, 2 or 3), number of midwives, regular use of mortality and morbidity reviews (MMRs) in the maternity unit. We also calculated a births/midwife ratio, defined as: [Number of births per year])/[average number of midwives working per 12-hour period in the delivery room].

### Ethics Statement

Our institutional review board (Comité de Protection des Personnes Ile de France Paris- XI) approved this study on September 13, 2012, as number 12066.

Participants were all midwives who completed a questionnaire about how they would respond to 2 clinical vignettes. By clicking on the survey link and completing the questionnaire, they provided informed consent to participate. Participants were informed about the purpose of the study at the beginning of the study (through the email that led them to contact the study site and by the introduction to the study).

### Role of the funding source

The study sponsor did not participate in the study design, data collection and analysis, decision to publish, or preparation of the manuscript. Authors had full access to all the data and had final responsibility for the decision to submit for publication.

### Statistical analysis

Data are available in the Table in S1 Table.

Due to the hierarchical structure of the data, with the midwives (first level) nested within maternity units (second level), we applied a two-level Poisson regression analysis. The outcome was the number of errors in adherence to guidelines. The first step was a univariate analysis with a two-level model for each independent factor with a random intercept at the maternity-unit level. In the second step, the variables for the midwives and the maternity units (that is, the staff and institutional variables) with a *P*-value < 0.20 in the univariate analysis were included in the multivariate two-level model. Interactions were tested for all the selected variables and kept when their *P*-value was <0.05.

To measure staff and institutional effects, we calculated incidence rate ratios (IRR) and their 95% confidence intervals (95%CI).

To estimate the general institutional effect, we calculated intraclass correlation coefficients (ICC) for error count. A small ICC value (close to 0) indicates that the maternity unit’s characteristics did not affect the error count.

All statistical tests were two-sided and *P*-values<0.05 were considered statistically significant. All models were fitted with the lme4 [39] package in R software [40] version 3.0.1.

## Results

We obtained complete responses from 450 midwives from 87 maternity-units (Fig 1).

Table 2 summarizes the characteristics of the midwives and their maternity units. All maternity units had a PPH protocol. For vignette 1, 113 (25.1%) midwives chose appropriate management that met all 14 criteria (0 errors), 230 (51.1%) at least 13 correct answers, and 315 (70.0%) at least 12 correct answers. For vignette 2, 19 (4.2%) midwives chose answers that met all 14 criteria (0 errors), 84 (18.6%) at least 13 correct answers, and 170 (37.7%) at least 12 correct answers.

**Table 2:** Characteristics of midwives and maternity units.

**Midwives**		**n = 450**
Gender: Female, n (%)		425 (94.4)
Age, year, mean (SD)		34.7 (8.4)
Experience, year, mean (SD)		11.4 (8.7)
Full-time job, n (%)		333 (74.0)
Level of risk taking *, n (%)	high or moderate	87 (19.6)
	low	357 (80.4)
Fear of malpractice *, n (%)	high or moderate	298 (67.1)
	low	146 (32.9)
**Maternity units**		**n = 87**
Status, n (%)	Public university	14 (16.1)
	Other public	51 (58.6)
	Private	22 (25.3)
Level of neonatal care, n (%)	Level 1	35 (40.2)
	Level 2	36 (41.4)
	Level 3	16 (18.4)
Number of births per year, mean (SD)		1623.2 (997.5)
Births/midwife ratio, mean (SD)		806.0 (212.0)
Mortality and morbidity reviews, n (%)		67 (77.0)

* missing data: n = 6 (1.3%)

### Factors associated with error count in PPH management

#### Univariate analysis

In vignette 1, midwives who were older, had more years of practice, and a higher risk-taking score had a significantly higher risk of error in adherence to guidelines. Conversely, both full-time practice and a higher ratio of births/midwife were significantly associated with a lower risk of error (Table 3). For vignette 2, again, midwives who were older or who had practiced longer were at higher risk of error, while two institutional factors, regular MMRs and a higher ratio of births per midwife, were both significantly associated with a lower risk (Table 3).

**Table 3:** Associations between error count of PPH management and staff and institutional factors. Univariate analysis for vignette 1 and vignette 2.

Staff and institutional factors	Vignette 1		Vignette 2	
	IRR [95% CI]	*P*-value	IRR [95% CI]	*P*-value
**Staff factors**				
Age (10 years)	1.19 [1.10; 1.30]	<0.001	1.14 [1.07; 1.21]	<0.001
Experience (10 years)	1.17 [1.08; 1.27]	<0.001	1.14 [1.07; 1.20]	<0.001
Full-time practice	0.82 [0.70; 0.96]	0.014	0.97 [0.86; 1.09]	0.594
Level of risk taking (high and moderate)	1.29 [1.09; 1.53]	0.004	0.99 [0.87; 1.13]	0.880
Fear of malpractice (high and moderate)	1.00 [0.86; 1.17]	0.959	0.91 [0.82; 1.02]	0.101
**Unit/institutional factors**				
Status		0.503	-	0.561
Public university	-			
Other public	1.15 [0.89; 1.48]		1.07 [0.90; 1.28]	
Private	1.04 [0.77; 1.41]		1.12 [0.91; 1.38]	
Level of neonatal care		0.699	-	0.082
Level 1	-			
Level 2	0.99 [0.79; 1.23]		0.94 [0.81; 1.09]	
Level 3	0.90 [0.69; 1.17]		0.82 [0.68; 0.98]	
Births/midwife ratio (100 births)	0.94 [0.90; 0.99]	0.010	0.96 [0.93; 0.99]	0.022
Mortality and morbidity reviews	0.86 [0.68; 1.08]	0.185	0.78 [0.67; 0.91]	0.002

#### Multivariate analysis

In vignette 1, after adjustment for all other factors selected in the univariate analysis and interactions, older age and a higher risk-taking score remained significantly associated with a higher risk of error in adherence to guidelines. Full-time practice remained significantly associated with a lower risk of error, and the performance of MMRs in the unit with a lower risk of error (IRR 0.80 [95%CI 0.64; 0.99], *P* = 0.037) (Table 4). We also observed a significant interaction between the births/midwife ratio and these reviews (IRR 0.85 [95%CI 0.75; 0.96], *P* = 0.011). At institutions with these reviews, each increase of 100 births/midwife decreased the risk of error by 7% (IRR 0.93 [0.89; 0.97], *P* = 0.001). Conversely, in maternity units without MMRs, each increase of 100 births/midwife increased the risk of error by 9%, although this difference was not significant (IRR 1.09 [95%CI 0.97; 1.22], *P* = 0.141).

**Table 4:** Associations between error count for PPH management and staff and institutional factors. Vignette 1 –multivariate analysis.

	IRR [95% CI]	*P*-value
**Staff factors**		
Age (10 years)	1.19 [1.09 ; 1.29]	<0.001
Full-time practice	0.83 [0.71 ; 0.97]	0.020
Level of risk taking (high and moderate)	1.41 [1.19 ; 1.67]	<0.001
**Unit/institutional factors**		
Mortality and morbidity reviews *	0.80 [0.64 ; 0.99]	0.037
Births/midwife ratio (100 births) if no MMRs *	1.09 [0.97 ; 1.22]	0.141
Births/midwife ratio (100 births) if MMRs *	0.93 [0.89; 0.97]	0.001

* Interaction between births/midwife ratio and MMRs: 0.85 [0.75; 0.96], *P* = 0.011

For vignette 2, after adjustment, older age remained significantly associated with a higher risk of error, and MMRs with a lower risk of error (IRR 0.78 [0.66; 0.93], *P* = 0.005) (Table 5). We again observed a significant interaction between the births/midwife ratio and MMRs (IRR 0.91 [0.82; 1.00], *P* = 0.042): at institutions with these reviews, each increase of 100 births/midwife decreased the risk of error by 4% (IRR 0.96 [0.92; 1.00], *P* = 0.027). Conversely, in the units without them, each increase of 100 births/midwife increased, albeit not significantly, the risk of error, by 6% (IRR 1.06 [0.97; 1.16], *P* = 0.224).

**Table 5:** Associations between error count for PPH management and staff and institutional factors. Vignette 2 –multivariate analysis.

	IRR [CI 95%]	*P*-value
**Personal factors**		
Age (10 years)	1.11 [1.05; 1.18]	<0.001
Fear of malpractice (high and moderate)	0.91 [0.82; 1.02]	0.100
**Unit/institutional factors**		
Level of neonatal care		0.906
Level 1	-	
Level 2	1.01 [0.87; 1.17]	
Level 3	0.97 [0.80; 1.18]	
Mortality and morbidity reviews *	0.78 [0.66; 0.93]	0.005
Births/midwife ratio (100 births) if no MMRs *	1.06 [0.97; 1.16]	0.224
Births/midwife ratio (100 births) if MMRs *	0.96 [0.92; 1.00]	0.027

* Interaction between births/midwife ratio and MMRs: 0.91 [0.82; 1.00], *P* = 0.042

The ICC for error count in vignette 1 was 0.09 [0.02; 0.18], meaning that 9% of the overall variation in the error count can be explained by the variation among maternity units. The ICC for error count in vignette 2 was 0.11 [0.04; 0.21].

## Discussion

### Main Findings

Older midwives and those at units that do not perform regular MMRs are significantly more likely to provide substandard care that fails to comply with guidelines. Part-time practice and a high risk-taking score were also associated with substandard care, but only in Vignette 1.

### Strengths and Limitations of the Study

To our knowledge, this is the first study showing the impact of the personal characteristics of obstetrics professionals (midwives’ age, full- vs part-time practice, risk-taking score) on the quality of PPH management. Our use of a methodology previously validated in the specific context of the emergency situation that is PPH [31] strengthens the internal validity of the study. Furthermore our sample of 450 midwives is large and the characteristics of participating midwives were similar to those of French midwives overall [41,42]; these points strengthen the external validity of the study.

However, our study has some limitations. Using case-vignettes is a theoretical approach: what we studied was midwives’ choices from a list of management options and not their actual practice. Three biases may overestimate the appropriateness of the management proposed by professionals: (i) the emergency and stress generated by PPH, which cannot be fully represented in the vignette; (ii) the likely social desirability bias; (iii) the multiple-choice format (compared, for example, with open questions), which might result in overestimating participant performance [43]. Despite these biases, we found complete adherence to guidelines to be fairly low.

We did not examine the protocols of all the participating units and thus do not know if they all actually incorporate the French guidelines in their protocols. Accordingly, when midwives made errors in adherence to guidelines, we could not know if 1) they were applying their unit’s protocol, but it did not comply with the national guidelines, or if 2) they failed to apply their protocol, which was consistent with national guidelines. This point may explain the observed center effect.

Because our study only included midwives, the generalization of the results may be possible only in countries where midwives provide initial management of PPH. Previous retrospective studies [25,26] have evaluated PPH cases managed by midwives and obstetricians together. The case-vignette method cannot transcribe a multidisciplinary approach. Midwives are not allowed, for example, to decide upon or perform surgery. They should, however, discuss the possibility of such treatment in collaboration with the obstetrician and anesthesiologist if the steps taken remain insufficient.

### Interpretation

It has already been demonstrated that physicians often fail to follow clinical practice guidelines, and lack of awareness and disagreement were suggested as possible barriers to adherence [44]. In our study, adherence was lower for vignette 2 than for vignette 1. Midwives may thus be uncomfortable about the optimal management of the PPH situation in vignette 2, possibly because it is less common.

The age of midwives was significantly associated with substandard care in both vignettes in our study. Possibly, older midwives intentionally made decisions that differ from guidelines because they considered their experience more relevant than guidelines or because they did not know the guidelines. Continuing training and internal team audit are therefore essential.

Only a few studies have examined the use of MMRs and found that they are associated with improved maternal mortality and morbidity [45,46,47].

Although the French national authority for health (“Haute Autorité de Santé”) has published recommendations to standardize the implementation of MMRs in institutions, we do not know if the units followed these recommendations for their MMRs. It is nonetheless interesting to observe that the multidisciplinary analysis involved in MMRs, regardless of their format, improve the quality of care.

Farquhar et al [23] also demonstrated the importance of organizational factors in a study of potentially avoidable maternal deaths in New Zealand, identified by an expert panel. Contributory factors were classified as organizational, staff-related, or environmental. Organizational factors were identified in 55% of all avoidable deaths, and in many cases more than one of these factors applied. Those most frequently identified were lack of protocols, inadequate education and training, and staff lack of knowledge and skills.

Multidisciplinary thinking and internal team audits are therefore essential for improving the quality of care.

## Conclusion

This case-vignette study allowed us to identify staff and institutional factors associated with substandard care in midwives’ management of PPH.

Our study identified MMRs as factors that could improve the quality of care. It appears especially necessary to implement MMRs in small maternity units and to encourage the participation of midwives.

As our study also demonstrated the influence of individual factors in the quality of PPH management, the reinforcement of continuous training at the individual level is a critical goal.

## Supporting Information

S1 File Vignette 1. (PDF)

S2 File Vignette 2. (PDF)

S1 Table database of midwives responses. (XLS)

S1 Text exhaustive list of possible answers to multiple-choice questions. (PDF)
