# Supplementary material for: Staff and Institutional Factors Associated with Substandard Care in the Management of Postpartum Hemorrhage
Source: PLoS One. 2016 Mar 24;11(3):e0151998. doi: 10.1371/journal.pone.0151998 (PMC4806984; doi:10.1371/journal.pone.0151998)
Supplement: S1 File — (PDF) [file pone.0151998.s001.pdf]

Vignette 1

Age : 33years
Gravida 2
Origin : french
BMI : 20

Antecedent : cesarean section 3 years earlier
Pregnancy : streptococcus B carriage
Term : 38 weeks of gestation
Spontaneous labor

Pre-operative blood test : haemoglobin: 10.2g/dL, platelet: 235G/L

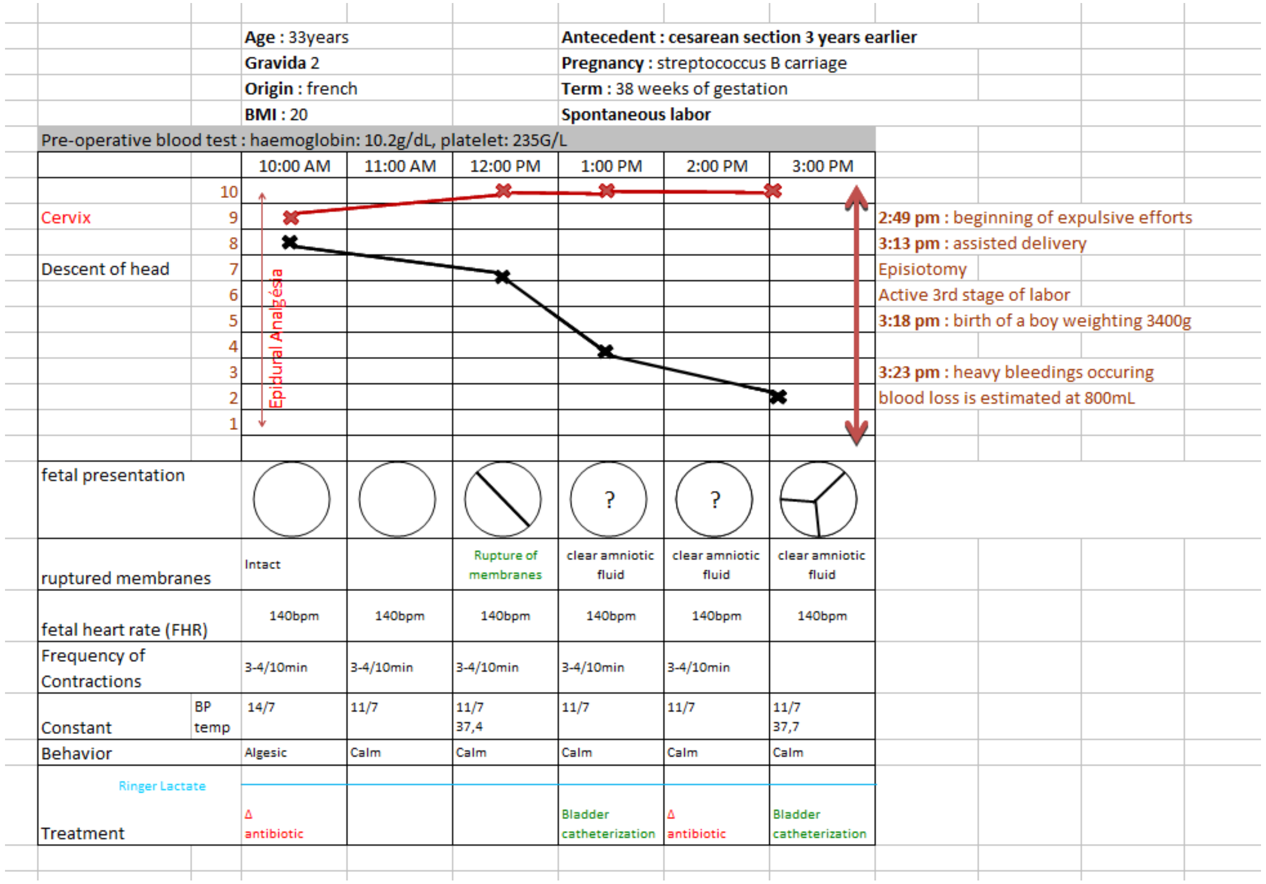

2:49 pm : beginning of expulsive efforts
3:13 pm : assisted delivery
Episiotomy
Active 3rd stage of labor
3:18 pm : birth of a boy weighting 3400g

3:23 pm : heavy bleedings occuring
blood loss is estimated at 800mL

		10:00 AM	11:00 AM	12:00 PM	1:00 PM	2:00 PM	3:00 PM
ruptured membranes		Intact		Rupture of membranes	clear amniotic fluid	clear amniotic fluid	clear amniotic fluid
fetal heart rate (FHR)		140bpm	140bpm	140bpm	140bpm	140bpm	140bpm
Frequency of Contractions		3-4/10min	3-4/10min	3-4/10min	3-4/10min	3-4/10min	
Constant	BP	14/7	11/7	11/7	11/7	11/7	11/7
	temp			37,4			37,7
Behavior		Algesic	Calm	Calm	Calm	Calm	Calm
Treatment	Ringer Lactate	Δ antibiotic			Bladder catheterization	Δ antibiotic	Bladder catheterization

What measures would you perform **within the next 15 minutes** ?

Vignette 1

Despite your actions, bleeding persists. Uterus is hypotonic

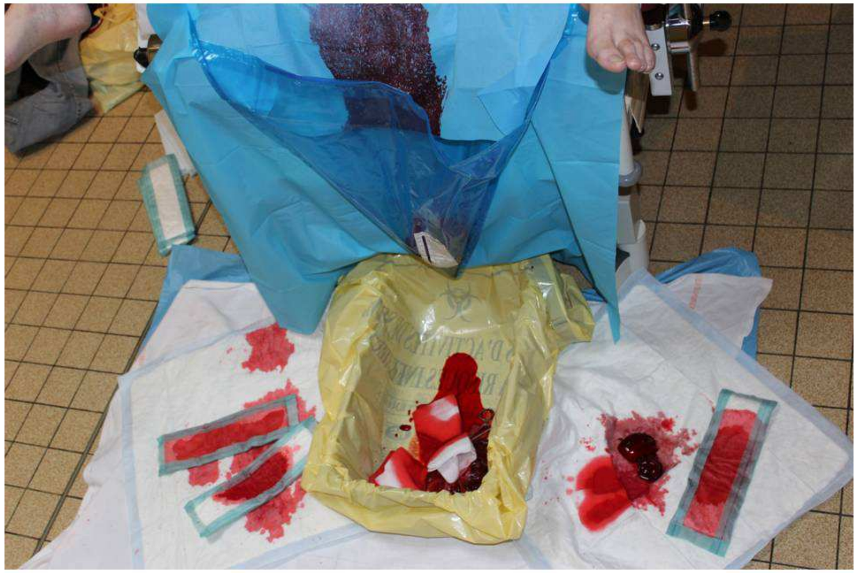

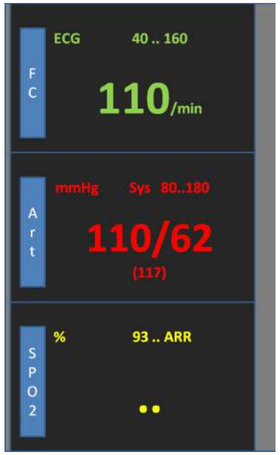

What measures would you perform **within the next 15 minutes** ?

Vignette 1

30 minutes later, despite your actions, bleedings persists. The uterus is hypotonic when you stop the uterine massage. The patient does not feel very well and is dizzy.

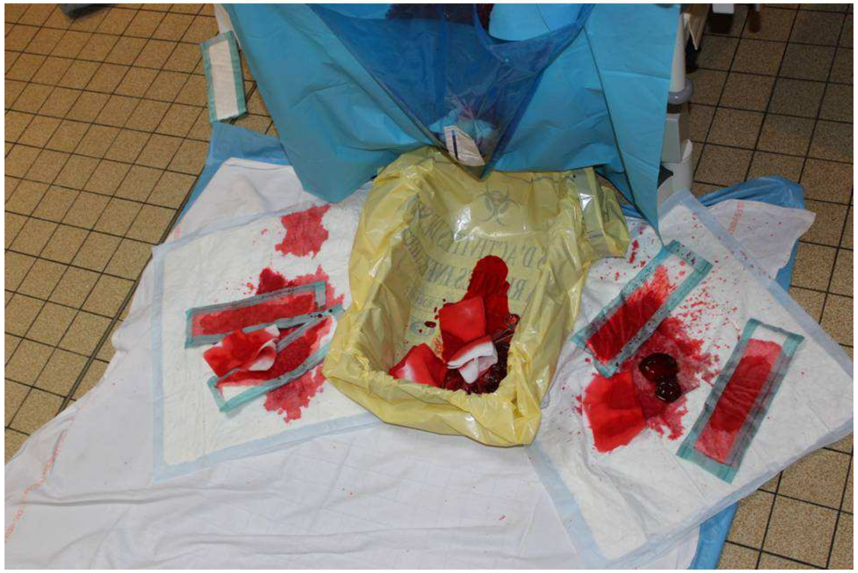

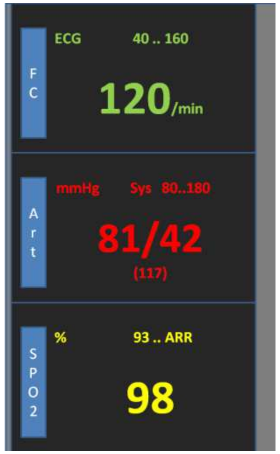

What measures you propose at this stage ?
