# Supplementary material for: Staff and Institutional Factors Associated with Substandard Care in the Management of Postpartum Hemorrhage
Source: PLoS One. 2016 Mar 24;11(3):e0151998. doi: 10.1371/journal.pone.0151998 (PMC4806984; doi:10.1371/journal.pone.0151998)
Supplement: S2 File — (PDF) [file pone.0151998.s002.pdf]

Vignette 2

**Age :** 31years	Course of pregnancy uneventful
**Gravida 1**	
**Origin :** Moroccan	**Term :** 40 weeks of gestation
**BMI :** 23	**Spontaneous labor**

Pre-operative blood test : haemoglobin: 13.1g/dL, platelet: 178G/L

Heure		8:00 PM	9:00 PM	10:00 PM	11:00 PM	12:00 AM	1:00 AM
	10						
Cervix	9						
	8						
Descent of head	7						
	6						
	5						
	4						
	3						
	2						
	1						

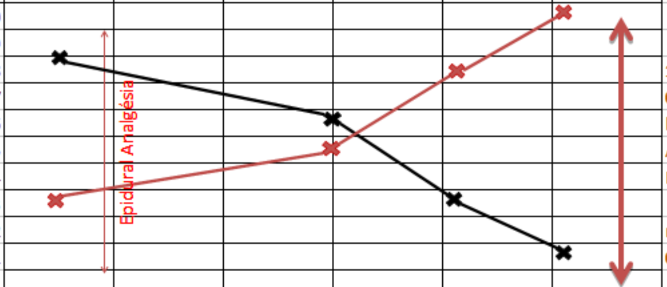

12:40 am: beginning of expulsive efforts
01:05 am : spontaneous vaginal delivery
boy weighting 3640g
Active 3rd stage of labor
Placenta delivered

minimal bleeding appears and stops with uterus massage
02:30 am : minimal bleeding persists, blood loss is estimated at 650mL

fetal presentation

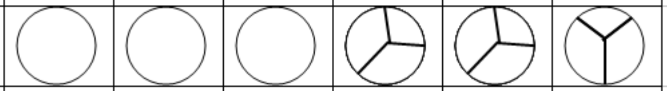

		8:00 PM	9:00 PM	10:00 PM	11:00 PM	12:00 AM	1:00 AM
ruptured membranes			Rupture of membranes	clear amniotic fluid	clear amniotic fluid	clear amniotic fluid	brown amniotic fluid
fetal heart rate (FHR)		140bpm	140bpm	140bpm	140bpm	160bpm	160bpm
Frequency of Contractions		4/10min	3-4/10min	3-4/10min	3-4/10min	4/10min	4/10min
Constant	BP	12/7	12/7	11/7	11/7	11/7	
	temp	36,9					
Behavior		Algic	Algic	Calm	Calm	Calm	Calm
Treatment	Ringer Lactate				Oxytocine		Bladder catheterization

What measures would you perform **within the next 15 minutes** ?

Vignette 2

Despite your actions, bleeding persists in thin stream. The uterus is tonic.

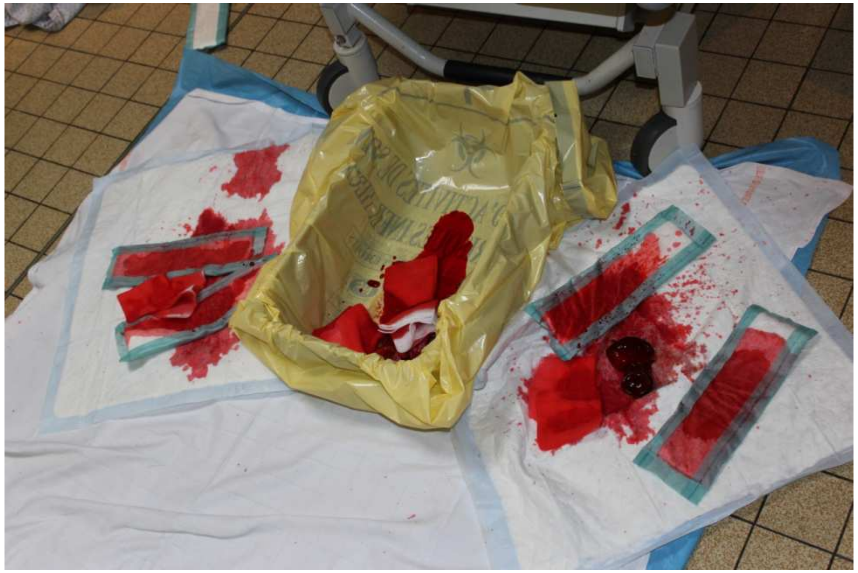

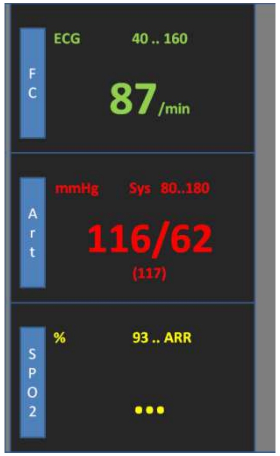

What measures would you perform **within the next 15 minutes** ?

Vignette 2

30 minutes later, despite your actions, bleeding is more abundant. The uterus is tonic.

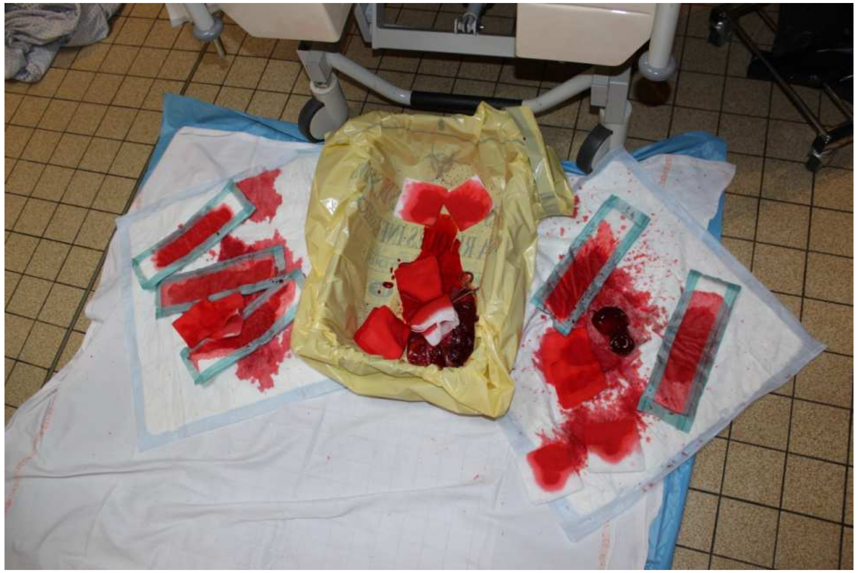

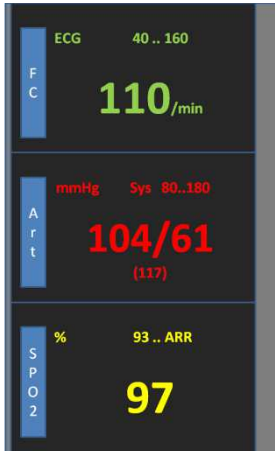

What measures you propose at this stage ?
