# Supplementary material for: Staff and Institutional Factors Associated with Substandard Care in the Management of Postpartum Hemorrhage
Source: PLoS One. 2016 Mar 24;11(3):e0151998. doi: 10.1371/journal.pone.0151998 (PMC4806984; doi:10.1371/journal.pone.0151998)
Supplement: S1 Text — (PDF) [file pone.0151998.s004.pdf]

Pharmacological management

□ Antibiotic

□ Cytotec® : misoprostol

□ per os	□ intrarectale	
□ 50 µg	□100 µg	□200 µg
□400 µg	□600 µg	□800 µg

□ Exacyl® : tranexamic acid intravenous

□ 500 mg	□ 1 g
□ 1.5 g	□ 2 g
□ 2.5 g	

□ Methylergometrine intramuscular

□ 0.1 mg	□ 0.2 mg

□ Naldor®: sulprostone intravenous for 1 h

□ 100 µg	□ 200 µg	□ 300 µg
□ 400 µg	□ 500 µg	□ 600 µg

□ Syntocinon®: oxytocin
- □ intravenous push (bolus):
  - □ 5 IU □ 10 IU □ 15 IU □ 20 IU
- □ slow intravenous (1min):
  - □ 5 IU □ 10 IU □ 15 IU □ 20 IU
- □ intravenous infusion:
  - □ 5 IU □ 10 IU □ 15 IU □ 20 IU
- □ flow acceleration of the current infusion

□ Other:

□ None

Non-pharmacological management

□ Surgical treatment considered

□ Bimanual uterine compression

□ Manual examination of the uterine cavity

□ Abdominal ultrasound

□ Selective arterial embolization considered

□ Uterine massage

□ Cervical examination with speculum

□ Perineal repair

□ Bladder catheterization

□ Intrauterine tamponade considered

□ Torsion of the cervix

□ Other:

□ None

Communication, monitoring and investigation

□ Alert other member of the team
- □ obstetric team
- □ anesthesiology team

□ Venipuncture for blood sampling
- □ coagulation factors
- □ liver function tests
- □ hemostasis
- □ serum electrolytes
- □ blood counts including platelet counts

□ Resuscitation
- □ second intravenous access
- □ monitoring
- □ volume replacement
- □ other :

□ Other:

□ None
